# Dissection of the mechanism of traditional Chinese medical prescription-Yiqihuoxue formula as an effective anti-fibrotic treatment for systemic sclerosis

**DOI:** 10.1186/1472-6882-14-224

**Published:** 2014-07-07

**Authors:** Ting Wu, Haiyan Chu, Wenzhen Tu, Mengmeng Song, Dongdong Chen, Jin Yuan, Ling Yu, Yanyun Ma, Qingmei Liu, Li Jin, Xiaodong Zhou, Hejian Zou, Wenyu Wu, Jiucun Wang

**Affiliations:** 1 National Ministry of Education Key Laboratory of Contemporary Anthropology, School of Life Sciences, Fudan University, Shanghai, PR China; 2 Department of Dermatology, Shanghai TCM-integrated Hospital, Shanghai, PR China; 3 Huashan Hospital, Shanghai, PR China; 4 Division of Rheumatology, University of Texas-Houston Health Science Center, Houston, USA; 5 Institute of Rheumatology, Immunology and Allergy, Fudan University, Shanghai, PR China; 6 International Network of Scleroderma Clinical Care and Research (InSCAR), Shanghai, PR China; 7 Department of Dermatology, Huashan Hospital, Shanghai, PR China

**Keywords:** Systemic sclerosis, Traditional Chinese Medicine, Fibrosis, Collagen, TGF-β

## Abstract

**Background:**

Systemic sclerosis (SSc) is a connective tissue fibrotic disease for which there is no effective treatment. Traditional Chinese Medicine (TCM), such as the Yiqihuoxue formula used in Shanghai TCM-integrated Hospital, has shown the efficacy of anti-fibrosis in clinical applications. This study was aiming to dissect the anti-fibrotic mechanism of Yiqihuoxue treatment for SSc.

**Methods:**

Bleomycin-induced mice and SSc dermal fibroblasts were treated with Yiqihuoxue decoction; NIH-3T3 fibroblasts were exposed to exogenous TGF-β1, and then cultured with or without Yiqihuoxue decoction. Luciferase reporter gene assay was used to determine the activity of Smad binding element (SBE). Quantitative reverse transcription-polymerase chain reaction (RT-PCR) was used to examine the mRNA levels of extracellular matrix (ECM) genes. The protein levels of type I collagen, Smad3 and phosphorylated-Smad3 (p-Smad3) were detected by western blotting. Student’s *t*-tests were used to determine the significance of the results.

**Results:**

Bleomycin-induced mice, SSc dermal fibroblasts and TGF-β1-induced NIH/3T3 fibroblasts showed higher levels of ECM gene transcriptions and collagen production. In addition, the phosphorylation level of Smad3 and activity of SBE were significantly increased after exogenous TGF-β1 induction. Whereas, Yiqihuoxue treatment could obviously attenuate fibrosis in bleomycin-induced mice, down regulate ECM gene expressions and collagen production in SSc dermal fibroblasts and TGF-β1-induced NIH/3T3 fibroblasts. Furthermore, the aberrantly high phosphorylation level of Smad3 and activity of SBE in the TGF-β1-induced NIH/3T3 fibroblasts were also dramatically decreased by Yiqihuoxue treatment.

**Conclusions:**

Yiqihuoxue treatment could effectively reduce collagen production via down-regulating the phosphorylation of Smad3 and then the activity of SBE, which are involved in the TGF-β pathway and constitutively activated in the progression of SSc.

## Background

Systemic sclerosis (SSc) is a connective tissue disease mainly characterized by extensive fibrosis in skin and internal organs, such as lung, heart, esophagus and kidney, among which skin fibrosis is the universal manifestation in SSc [1,2]. Previous reports have shown that SSc confers a mortality risk and fibrosis of internal organs is the leading cause of death in SSc patients [3,4]. Until now, the precise mechanisms of skin fibrosis in SSc remain unclear, and there are no generally accepted and effective medical treatments for fibrosis.

Fibrosis is usually caused by the excessive production, deposition and contraction of extracellular matrix (ECM) components, especially collagen [5]. Fibroblast is the principal cell type responsible for turnover and composition of ECM. The “activated” fibroblast and its contractile and secretory counterpart, myofibroblast, are the primary cell types responsible for the persistent production and deposition of ECM. Tissue injury initiates the chronic inflammation generally involving the activation of inflammatory and immune cells which secrete cytokines, chemokines and growth factors. Then resident fibroblasts (quiescent fibroblasts), pericytes, fibrocytes, epithelial and endothelial cells are recruited, activated and finally differentiated into myofibroblasts. The “activated” fibroblast is an intermediate stage between resident fibroblast and myofibroblast. In addition, the “activated” fibroblast and myofibroblast also produce cytokines and growth factors such as TGF-β and CTGF to support further fibrogenesis [6-8].

TGF-β is an important regulator in collagen production, and normal fibroblasts stimulated by TGF-β displayed features of SSc fibroblasts [9,10]. Previous research suggested that the aberrant expression of TGF-β led to the activation of ECM synthesis and dysfunction of ECM degradation simultaneously [11], resulting in the unbalance between ECM production and degradation and thus fibrosis. Although the precise mechanism of fibrosis in SSc remains unknown, those results suggest a potential clue to uncover it. Latent TGF-β is cleaved to release active TGF-β, then TGF-β binds to the type II TGF-β receptor (TGFBRII) and activates the type I TGF-β receptor (TGFBRI). Activated TGFBRI transduces signals to the Smad proteins, that is, phosphorylates Smad3. The phosphorylation of Smad3 allows it to form a heterodimer with Smad4, which translocates into nucleus and interacts with Smad binding element (SBE) in the promoter origin of the target genes, resulting in the induction of gene expressions including ECM genes [12-14]. Smad3 seems to be a key signal transducer involved in the Smad-dependent TGF-β signaling pathway. Indeed, Smad3-deficient mice exhibited attenuated lung fibrosis induced by bleomycin [15], and overexpression of Smad3 significantly increased the activity of type I collagen promoter [16].

CTGF is a key effector in the downstream of TGF-β and plays a role in the regulation of fibroblast proliferation and migration as well as TGF-β-dependent ECM production [17]. In addition, *Ctgf* siRNA also ameliorated fibrosis in skin and reduced inflammation in lungs of bleomycin-induced mice [18]. SPARC, secreted protein, acidic and rich in cysteine, is one of the major components in ECM. Elevated expression of SPARC has been reported in numerous animal models of fibrotic diseases and human fibrotic tissues [19]. Furthermore, our previous work and others’ also showed *SPARC* siRNA effectively reduced fibrosis in SSc dermal fibroblasts, TGF-β1-stimulated fibroblasts, skin and lung tissues from bleomycin-induced mice [18,20,21].

It is thorny and challenging to find an effective therapy for SSc, because of its complicated interaction of vascular, immunologic and fibrotic components. Traditional Chinese medicine (TCM) has a long history of dealing with diseases for several thousand years. More importantly, TCM with combinations of various components and multiple drug targets shows an overwhelming advantage of treating complicated diseases, such as acute promyelocytic leukemia (APL) [22]. Currently, TCM has been applied to treat SSc patients in many hospitals in China. The prescription of Yiqihuoxue formula provided by the Shanghai TCM-integrated Hospital has been shown a satisfactory capability of attenuating SSc-associated fibrosis in clinical applications. The two main constituents of Yiqihuoxue formula are *Astragalus membranaceus* and *Salvia miltiorrhiza*, which have been proven effective in treating diseases. Compound *Astragalus* and *Salvia miltiorrhiza* extract could attenuate liver fibrosis induced by carbon tetrachloride via inhibiting Smad2 phosphorylation and α-SMA expression [23]; a standardized extract from *Paeonia lactiflora* and *Astragalus membranaceus* exerted an anti-fibrotic effect in rats induced by porcine serum via down-regulating PDGFR-β, inhibiting HSC proliferation and MAPK activation [24]; constituents of *Radix Salviae Miltiorrhizae* inhibited proliferation and procollagen synthesis in SSc dermal fibroblasts [25]; one of the main active components from *Radix Salviae miltiorrihizae* called Salvianolic Acid B could attenuate liver fibrosis by inhibiting Angiotensin II signaling [26]. In order for the modernization and generalization of Yiqihuoxue formula in treating SSc, the precise anti-fibrotic mechanism remained to be addressed by using molecular approaches.

Aiming to explore the underlying mechanism of TCM in treatment of SSc-associated fibrosis, we used the treatment of Yiqihuoxue formula *in vivo* and *in vitro* in the present study.

## Methods

### Composition and preparation of Yiqihuoxue formula

Yiqihuoxue formula was obtained from the Shanghai TCM-integrated Hospital and the full components are *Astragalus membranaceus, Salvia miltiorrhizae, Angelica sinensis, Caulis spatholobi, Semen persicae, Tuyuan, Agkistrodon piscivorus, Centella asiatica, Ganoderma lucidum, Herba epimedii, Poria peel and Radix glycyrrhizae*, of which *Astragalus membranaceus* and *Salvia miltiorrhiza* are two major components. The Yiqihuoxue decoction was boiled with ultrapure water as the doctor’s directions. In *in vivo* study, 250 μl Yiqihuoxue decoction was fed to mice twice a day at the concentration of 23 g/d/kg. In *in vitro* study, it was filtered through a filter membrane in 0.22 μm and diluted to the concentration of 10 mg/ml in Dulbecco’s modified Eagle’s medium (DMEM, Invitrogen, Carlsbad, CA, USA) with 10% fetal calf serum (FCS, Invitrogen).

### Cell culture

Skin biopsy specimens were obtained from three diffuse SSc patients, who were fulfilled the American College of Rheumatology (formerly, the American Rheumatism Association) criteria for SSc [14]. Skin biopsy specimens were collected from three normal controls who had no history of autoimmune and other dermal diseases. All patients provided written contents, and the study was approved by the School of Life Sciences, Fudan University, China.

Skin samples were transported in DMEM supplemented with 10% FCS for processing the same day. The skin samples were washed in 75% ethanol, phosphate buffered saline (PBS), and DMEM with 10% FCS. Cultured fibroblast strains were established by mincing tissues and placing them into 60-mm culture dishes secured by glass coverslips. Third- to fifth-passage human dermal fibroblasts were placed into 12-well culture plates at the density of 1 × 10^5^ cells per well for gene and protein expression assays.

### Bleomycin-induced dermal fibrosis mouse model establishment

Specific pathogen-free (SPF) mice on a C57BL/6 background were purchased from Sino-British Sippr/BK Lab Animal Ltd. (Shanghai, China). Skin fibrosis was induced in 7-week-old female mice by administering local injections of bleomycin for 3 weeks. Briefly, bleomycin dissolved in filter-sterilized saline at a concentration of 200 μg/ml, and 100 μl of bleomycin was administered daily by subcutaneous injection into defined areas of 1 cm^2^ on the upper back. Subcutaneous injections of 100 μl saline were used as controls. After 3 weeks, the mice were killed by chloral hydrate anesthesia and skin tissues were harvested in order to analyze gene and protein expression levels. All the animal protocols were approved by the School of Life Sciences, Fudan University, China.

### Drug administration regimens

**Figure 1:**
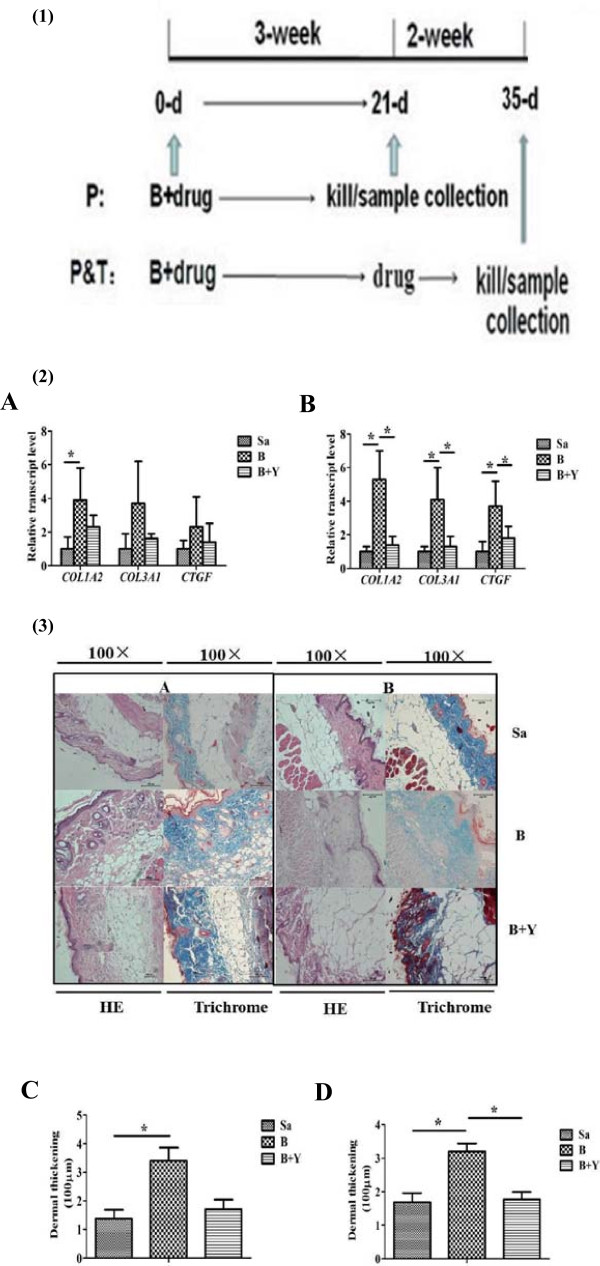
**Gene expressions and examination of skin tissues from bleomycin-induced mice with different treatments. (1)** Drug administration regimens for mouse model establishment. B, bleomycin; P, prevention group; P & T, prevention & treatment group. **(2)** Relative transcript levels of *Col1a2*, *Col3a1* and *Ctgf* in skin tissues from bleomycin-induced mice with different treatments in P and P & T group. Sa, saline; B, bleomycin; Y, Yiqihuoxue decoction. **A**. P group; **B**. P & T group. The expression level of each gene in saline-treated mice was normalized to 1. *, *P* <0.05; **, *P* <0.001. **(3)** Representative histological analysis of HE and Masson’s trichrome staining of mouse skin tissues with different treatments in high magnifications (100 ×). Four mice were used for each group. **A**. P group; **B**. P & T group; **C**, **D**, results of analysis of dermal thickening from skin tissues from mouse models with different treatments in P and P & T group, respectively. Bars showed the mean ± SD results of analysis of four mice. *, *P* <0.05; **, *P* <0.001.

Mice were randomly divided into two groups. As described in Figure 1(1), the first group (Prevention, P) received Yiqihuoxue decoction daily at the same time as bleomycin administration for 3 weeks. The second group (Prevention & Treatment, P & T) received Yiqihuoxue decoction for another 2 weeks compared with the first group. Four mice were used in each group.

### Preparation and treatment of recombinant TGF-β1 and TCM

Recombinant TGF-β1 was purchased from R&D Systems (Minneapolis, MN, USA) and diluted to the concentration of 10 ng/μl in DMEM medium with 1% FCS. SSc dermal fibroblasts were placed into a 12-well culture plate at the density of 1 × 10^5^ cells per well and cultured for 24 hours followed by treatment with or without Yiqihuoxue decoction (10 mg/ml). These cells were then examined 24 hours later for gene and protein expressions. NIH/3T3 fibroblasts were also seeded at the same density as SSc fibroblasts and cultured with or without TGF-β1 (10 ng/μl) for 24 hours after starved overnight. Then the culture medium was replaced by the normal medium with or without Yiqihuoxue decoction (10 mg/ml) and examined 24 hours later for gene and protein expressions.

### Luciferase reporter gene assay

pGL3-SBE4-Luc was a gift from Dr. Kiyoshi Higashi (Sumitomo Chemical Co., Ltd., Osaka, Japan), which consisted of four short tandem repeats of Smad binding element (SBE) (GTCTAGAC) with a minimal promoter (TATA box).The plasmid of pRL-SV40 was used as an internal control. NIH/3T3 fibroblasts were placed into a 24-well culture plate, and then 500 ng of pGL3-SBE4-Luc or pGL3-Basic plasmid were co-transfected with 10 ng of pRL-SV40 per well using Lipofectamin 2000 (Invitrogen) according to the manufacturer’s protocol. After 6 hours’ transfection, the culture medium was replaced by TGF-β1 medium with or without Yiqihuoxue decoction. Cell lysates were harvested 24 hours later and luciferase activity assay was performed according to the manufacturer’s protocol of Dual-luciferase reporter assay system (Promega, Madison, WI, USA) with a GloMax 20/20 Luminometer (Promega).

### Preparation and phosphorylation assay

Antibodies to Smad3 and p-Smad3 were purchased from Cell Signaling Technology Inc. (Beverly, MA, USA). NIH/3T3 fibroblasts were placed into a 12-well culture plate at the density of 1 × 10^5^ cells per well and grown until confluency. The culture medium was added by Yiqihuoxue decoction and cultured for 24 hours. After that, NIH/3T3 fibroblasts were treated with TGF-β1 for 1 hour, and cell lysates were harvested for western blotting.

### Quantitative reverse transcription-polymerase chain reaction (RT-PCR)

RT-PCR was performed using an ABI Prism 7900 Detector Sysyem (Applied Biosystems, Foster City, CA, USA). The specific primers for each gene were designed using Primer 5 and synthesized by Generay Biotech Co., Ltd. (Shanghai, China). Total RNA samples were extracted from the cultured fibroblasts using TRIzol reagent (Invitrogen). Complementary DNA (cDNA) was synthesized using High Capacity cDNA Reverse Transcription Kit (Applied Biosystems). RT-PCR was performed using SYBR Premix ExTaq from TaKaRa Biotechnology Co., Ltd (Dalian, China). The data obtained from the assays were analyzed with SDS 2.3 software (Applied Biosystems). The amount of total RNA in each sample was normalized with *GAPDH*/*Gapdh* transcription levels.

### Western blotting analysis

The protein concentration was determined using BCA protein assay kit (Beyotime Institute of Biotechnology, Shanghai, China). Equal amount of proteins from each sample was subject to sodium dodecyl sulfate-polyacrylamide gel electrophoresis (SDS-PAGE) on a 10% polyacrylamide gel. Resolved proteins were transferred onto a polyvinylidenedifluoride (PVDF) membrane (Millipore, Billerica, MA, USA), and then blocked for 1 hour at room temperature using Tris Buffered Saline Tween (TBST) with 5% Bull Serum Albumin (BSA). After that, blotted proteins were incubated at 4°C overnight with a 1:1000 dilution of anti-mouse collagen antibody (Millipore), anti-Smad3 antibody (Cell Signaling Technology), and anti-p-Smad3 antibody (Cell Signaling Technology), as well as 1:500 dilution of anti-human collagen antibody (Millipore), respectively. Anti-GAPDH antibody (Cell Signaling Technology) was used as an internal control. After three washes with TBST for 30 minutes, the blotted proteins were incubated with the secondary antibody for 1 hour at room temperature, which was horse-radish peroxidase-conjugated anti-mouse, anti-rabbit or anti-goat IgG. Specific proteins were detected using an enhanced chemiluminescence system (Thermo Fisher Scientific Inc., Waltham, MA, USA), and the intensity of bands was quantified using ImageQuantTL software (General Electric Company, Fairfield, CT, USA).

### Histological analysis

For the assessment of histopathological changes, skin tissues were embedded in paraffin after fixed with 4% paraformaldehyde. Then 4 um-thick skin sections were stained with hematoxylin/eosin or Masson’s trichrome staining for better visualization of the tissue structure. Dermal thickness was analyzed with a Nikon Eclipse 80i microscope (Nikon, Badhoevedorp, The Netherlands) by measuring the maximal distance between the epidermal–dermal junction and the dermal–subcutaneous fat junction at 4 different skin sections in each mouse, as previously described [27]. Hypodermal thickness was determined by measuring the thickness of the subcutaneous connective tissue beneath the panniculus carnous at 4 different sites of the upper back in each mouse. The evaluation was performed by 2 independent examiners.

## Results

### Yiqihuoxue treatment ameliorated skin fibrosis in bleomycin-induced mouse models

**Figure 2:**
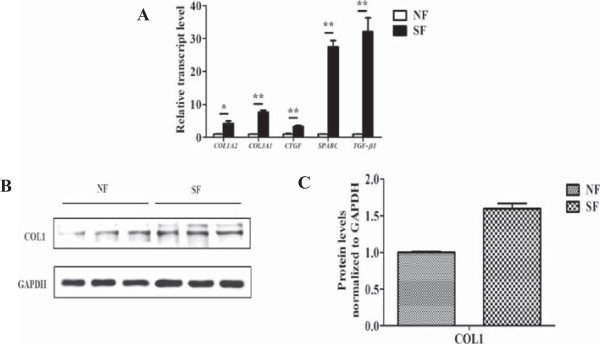
**Gene expressions and collagen production between SSc dermal fibroblasts and normal controls. (A)** Relative transcript levels of *COL1A2*, *COL3A1*, *CTGF*, *SPARC* and *TGF-β1* in three SSc dermal fibroblasts and normal controls. The expression level of each gene in normal controls was normalized to 1. *, *P* <0.05; **, *P* <0.001. SF, SSc dermal fibroblast; NF, normal dermal fibroblast. **(B)** Western blotting analysis of type I collagen (COL1) in the three SSc dermal fibroblasts and normal dermal fibroblasts. **(C)** Densitometric analysis of western blotting for type I collagen (COL1). Bars showed the mean ± SD results of three paired fibroblast strains (normal and SSc). *, *P* <0.05; **, *P* <0.001.

In this study, bleomycin-induced skin fibrosis mouse models were used to evaluate the anti-fibrotic effect of TCM treatment. Mice were randomly divided into two groups as described in Figure 1(1): Prevention (P) group and Prevention & Treatment (P & T) group. As measured by quantitative real-time RT-PCR, the transcripts of *Col1a2*, *Col3a1*, and *Ctgf* in both groups showed increased expressions in the skin tissues from bleomycin-induced mice, and Yiqihuoxue treatment recovered them nearly to the normal levels (Figure 1(2)). Moreover, the most significant effects of bleomycin and Yiqihuoxue treatment were detected in the P & T group, i.e., the fold changes of each gene in the skin tissues from bleomycin-induced mice were 5.3 ± 1.7-folds for *Col1a2* (*P* = 0.016), 4.1 ± 0.4-folds for *Col3a1* (*P* = 0.037), and 3.7 ± 1.5-folds for *Ctgf* (*P* = 0.007), respectively. After Yiqihuoxue treatment, significant reductions of *Col1a2* (74.2% with *P* = 0.020), *Col3a1* (67.1% with *P* = 0.031), and *Ctgf* (49.9% with *P* = 0.037) were found (Figure 1-2B). HE stain of skin tissues (Figure 1(3)) showed a significant dermal thickening after the induction of bleomycin in P & T group (*P* = 0.028), compared with the saline injection group. After Yiqihuoxue treatment, dermal thickening was notably improved (*P* = 0.024). Attenuation of dermal thickening also was found in P group after drug administration, though not very significant. Masson’s trichrome (Figure 1(3)) results were consistent with those of HE stain. Therefore, Yiqihuoxue treatment could significantly attenuate fibrosis and displayed an anti-fibrotic role in the treatment of SSc. More interestingly, Yiqihuoxue treatment not only exerted a powerful therapeutic action, but also presented a potential preventive effect in treating SSc.

### Elevated expressions of ECM genes and excessive collagen production in cultured fibroblasts from the skin tissues of patients with SSc

Fibroblasts play a primary role in the collagen production of SSc. RT-PCR data showed ECM genes including *COL1A2*, *COL3A1*, *CTGF*, *SPARC* and *TGF-β1* showed constitutive over-expressions in the SSc dermal fibroblasts compared with those in the healthy controls (Figure 2A). The fold changes of each gene in the SSc fibroblasts were 3.1 ± 0.85-folds (*P* = 0.003) for *COL1A2*, 6.7 ± 0.59-folds (*P* < 0.001) for *COL3A1*, 2.4 ± 0.28-folds (*P* < 0.001) for *CTGF*, 26.4-folds ± 1.91 (*P* < 0.001) for *SPARC* and 31.1 ± 4.19-folds (*P* < 0.001) for *TGF-β1*, respectively. Western blotting analysis showed a similar tendency of higher protein levels of type I collagen. Thus, fibroblasts were highly activated and synthesized abundant collagen proteins during the progress of SSc.

### Yiqihuoxue treatment reduced type I collagen production in the cultured fibroblasts from the skin tissues of patients with SSc

**Figure 3:**
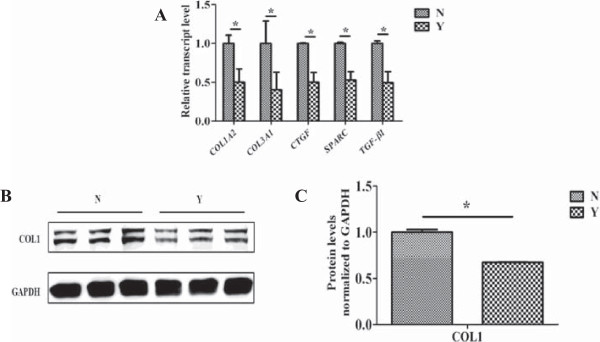
**Gene expressions and collagen production between SSc dermal fibroblasts with or without Yiqihuoxue treatment. (A)** Relative transcript levels of *COL1A2*, *COL3A1*, *CTGF*, *SPARC* and *TGF-β1* in three SSc dermal fibroblasts with or without Yiqihuoxue treatment. N, non-treated; Y, Yiqihuoxue decoction. The expression level of each gene in non-treated group was normalized to 1. *, *P* <0.05; **, *P* <0.001. **(B)** Western blotting analysis of type I collagen (COL1) in the three SSc dermal fibroblasts with or without Yiqihuoxue treatment. **(C)** Densitometric analysis of western blotting for type I collagen (COL1). Bars showed the mean ± SD results of three assays. *, *P* <0.05; **, *P* <0.001.

As shown in Figure 3A, significant reductions of *COL1A2* (49.8% with *P* = 0.012) and *COL1A3* (59.6% with *P* = 0.047) were observed after Yiqihuoxue treatment. In addition, other ECM genes including *CTGF*, *SPARC* and *TGF-β1* were also down-regulated by Yiqihuoxue treatment. Results of western blotting (Figure 3B, 3C) also showed a significant reduction of type I collagen with Yiqihuoxue treatment (32.6% with *P* = 0.040). Thus, Yiqihuoxue treatment could attenuate fibrosis by weakening the capacity of collagen production in fibroblasts.

### Yiqihuoxue treatment attenuated type I collagen production stimulated by exogenous TGF-β1 in the NIH/3T3 fibroblasts

**Figure 4:**
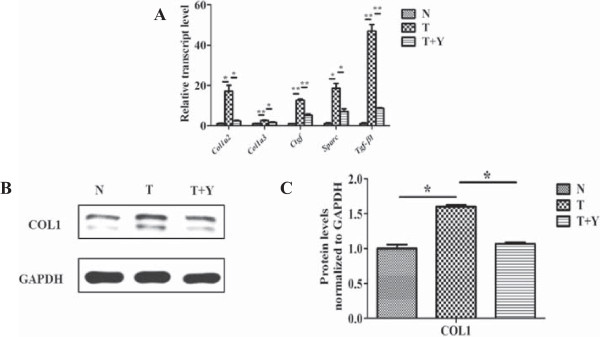
**Gene expressions and collagen production in NIH/3T3 fibroblasts with or without TGF-β1 and/or Yiqihuoxue treatment. (A)** Relative transcript levels of *Col1a2*, *Col3a1*, *Ctgf*, *Sparc* and *Tgf-β1* in murine 3T3 fibroblasts with different treatments. N, non-treated; T, TGF-β1; Y, Yiqihuoxue decoction. The expression level of each gene in non-treated murine 3T3 fibroblasts was normalized to 1. *, *P* <0.05; **, *P* <0.001. **(B)** Western blotting analysis of type I collagen (COL1) in murine 3T3 fibroblasts with different treatments. **(C)** Densitometric analysis of western blotting for type I collagen (COL1). Bars showed the mean ± SD results of three assays. *, *P* <0.05; **, *P* <0.001.

To clarify the mechanism of anti-fibrotic efficacy in Yiqihuoxue treatment *in vitro*, we utilized the cell line of NIH/3T3 fibroblasts to establish an *in vitro* model in this study. Results of RT-PCR (Figure 4A) showed that the transcript levels of *Col1a2* and *Col3a1* were dramatically increased (16.1 ± 2.99-folds and 1.7 ± 0.14-folds, respectively) after the stimulation of exogenous TGF-β1, and exhibited an SSc-like phenotype. In parallel, the transcript levels of *Ctgf*, *Sparc* and *Tgf-β1* were also highly elevated by 10.7 ± 1.21-folds to 46.0 ± 3.27-folds, respectively. However, Yiqihuoxue treatment attenuated fibrosis induced by exogenous TGF-β1 and restored those gene expressions almost to the normal levels. Western blotting analysis also indicated that type I collagen production was highly activated by exogenous TGF-β1 (60.0% increase with *P* = 0.022), while decreased by 33.2% (*P* = 0.002) after TCM treatment (Figure 4B, 4C).

### Yiqihuoxue treatment attenuated type I collagen production via down-regulating the phosphorylation level of Smad3 and then the activity of SBE in the type I collagen promoter

**Figure 5:**
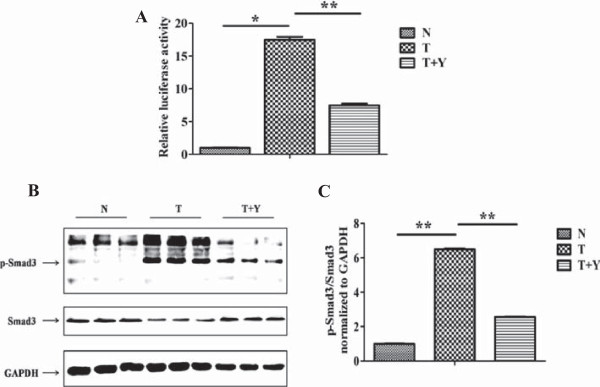
**Relative activity of SBE and phosphorylation of Smad3 in NIH/3T3 fibroblasts with different treatments. (A)** Relative activity of SBE in the region of type I collagen promoter in murine 3T3 fibroblasts with different treatments. N, non-treated; T, TGF-β1; Y, Yiqihuoxue decoction. The relative activity of SBE in non-treated murine 3T3 fibroblasts was normalized to 1. *, *P* <0.05; **, *P* <0.001. **(B)** Western blotting analysis of p-Smad3 and Smad3 in murine 3T3 fibroblasts with different treatments. p-Smad3, phosphorylated-Smad3. **(C)** Densitometric analysis of western blotting for p-Smad3 and Smad3. Bars showed the mean ± SD results of three assays. *, *P* <0.05; **, *P* <0.001.

Numerous researches have revealed that TGF-β signaling pathway plays a pivotal role in the fibrogenesis of SSc [28]. Furthermore, Smad binding element (SBE) is located in the type I collagen promoter, which mediates the transcriptional up-regulation stimulated by TGF-β together with the adjacent Sp-1 binding site [29]. To clarify the mechanism by which Yiqihuoxue treatment reduced collagen production, we conducted luciferase reporter gene assay. As shown in Figure 5A, the activity of SBE in the type I collagen promoter significantly increased by 19.8 ± 1.49-folds (*P* = 0.031) after the stimulation of exogenous TGF-β1, however, Yiqihuoxue treatment restored it nearly to the normal level (*P* < 0.001). Smad proteins, particularly Smad3, are considered as important signal transducers in the TGF-β signaling pathway. Moreover, the phosphorylation of Smad proteins could transduce signals and activate gene transcriptions in the downstream of TGF-β signaling pathway. To further investigate whether the phosphorylation of Smad3 contributed to the reduction of SBE activity, this study subsequently detected the phosphorylation levels of Smad3 after the stimulation of exogenous TGF-β1 with or without Yiqihuoxue treatment. As illustrated in Figure 5B and C, p-Smad3 was remarkably increased after the stimulation of exogenous TGF-β1 (5.5 ± 0.05-folds with *P* < 0.001), however, a lower phosphorylation level of Smad3 appeared after Yiqihuoxue treatment (60.5% decrease with *P* =0.001). Consequently, Yiqihuoxue treatment reduced collagen production via decreasing the phosphorylation level of Smad3 and inhibiting the interaction of Smad3/4 complex with SBE in the type I collagen promoter.

## Discussion

SSc is a typical chronic fibrotic disease with multiple-system involvement, mainly including skin and various visceral organs such as lung, heart, esophagus and kidney. Its etiology remains to be unveiled so far. Currently, principal medical therapies for SSc are still restricted to certain immunomodulatory and anti-fibrotic agents, such as glucocorticoid, colchicine and cyclophosphamide, which are used to treat symptoms of SSc with limited therapeutic effects. What is worse, side effects of these medications undoubtedly add to the burden of patients’ lives in general. Applications of TCM to treat diseases have a long history in China. Based on compatible principles of Chinese Medicine and symptoms of patients, TCM formula is usually composed of several herbs or minerals, which can exert a comprehensive effect on the diseases. Although working slowly, it is mild and less harmful to the quality of patients’ lives. In China, TCM treatments have been applied to treat SSc patients by many hospitals, among which Yiqihuoxue formula is a good example and has been proven an anti-fibrotic efficacy in clinical applications. Therefore, it is imperative to gain a better understanding of the mechanism of TCM as an effective anti-fibrotic treatment for SSc.

In the progress of SSc, vascular dysfunction, autoimmunity and inflammation may jointly contribute to the fibrogenesis [6]. *Salvia miltiorrhiza* is one of the main herbs in Yiqihuoxue formula, and its active component, salvianolic acid, has the function of removing blood stasis to improve vascular dysfunction [30,31]. Several researches indicated that salvianolic acid B (SAB) could attenuate liver fibrosis via TGF-β-related signaling pathways [32,33], and SAB addition inhibited Smad3 protein expression and its nuclear translocation in hepatic stellate cells [34]. In this study, we also found that Yiqihuoxue treatment could significantly reduce the phosphorylation level of Smad3 and the activity of SBE (Figure 5), so that we assumed that *Salvia miltiorrhiza* in Yiqihuoxue formula exerts its anti-fibrotic effect via Smad-dependent TGF-β signaling pathway. In addition, *Astragalus membranaceus* is also one of the main components in Yiqihuoxue formula, and useful for immune enhancement and anti-inflammation [35,36]. *Astragalus membranaceus* has been used in TCM for thousands of years. In modern Chinese Medicine, it has been used for cardiovascular and immunity system improvement [37,38]. The related constituents from *Astragalus membranaceus* exhibited an anti-inflammatory effect by the inhibitory activity of NF-kB [39], whose inhibition could also down-regulate the expression of CTGF in SSc fibroblasts [40]. Here, we proposed that *Astragalus membranaceus* attenuated fibrosis because of its immunomodulatory and anti-inflammatory properties. However, the precise mechanism of TCM treatment for vascular and immune system improvement remains unclear and deserves further efforts.

More interestingly, we also found that the absence of the two animal products in Yiqihuoxue formula, *Tuyuan* and *Agkistrodon piscivorus*, had no effects on decreasing SBE activity and Smad3 phosphorylation in the TGF-β1-treated NIH/3T3 fibroblasts (Additional file 1: Figure S1), indicating that these two animal components were not the necessary parts of the treatment formulation for systemic sclerosis, which meant that Yiqihuoxue formula had some redundancies. Thus, studies on the isolation of active ingredients and optimization of treatment formulation will greatly benefit the broader prospect for Yiqihuoxue formula and other traditional Chinese medical treatment.

Fibrosis is the main and most universal manifestation of SSc, which is caused by the unbalanced state between ECM production and degradation. TGF-β is considered as a potent profibrotic cytokine involved in the pathogenesis of SSc, because of its fibrotic functionality of stimulation of ECM synthesis and inhibition of metalloproteinase production [13]. Type I and type III collagens are two major proteins in the ECM components. CTGF is an important downstream cytokine which is regulated by TGF-β and has a synergistic effect with TGF-β [41,42]. In this study, overexpressions of both type I and type III collagen, and other ECM genes such as *CTGF*/*Ctgf*, *SPARC*/*Sparc*, *TGF-β1*/*Tgf-β1* were observed in bleomycin-induced mice, SSc dermal fibroblasts as well as TGF-β1-induced cell model (Figures 1, 2, 4), however, Yiqihuoxue treatment could recover them to the normal levels (Figures 1, 3, 4). Notably, the effects of Yiqihuoxue formula on the bleomycin-treated mice were more significant in the Prevention & Treatment group compared with those in the Prevention group (Figure 1), both in the reduction of ECM genes (*Col1a2*/*Col3a1*/*Ctgf*) by TCM and histopathological changes. It suggested that the effect of Yiqihuoxue formula was much better when administrated for long-term treatment, and it might exert its anti-inflammatory function in the early stage and anti-fibrotic function in the later stage.

## Conclusions

Our studies preliminarily demonstrated that Yiqihuoxue treatment for SSc significantly reduced collagen production in both *in vivo* bleomycin-induced mouse models and *in vitro* SSc dermal firbroblasts and TGF-β1-induced NIH/3T3 fibroblasts, via down-regulating the phosphorylation of Smad3 and the activity of SBE in the type I collagen promoter. Although our studies were limited to mouse model and cell culture conditions, it was the first attempt to explore the mechanism of TCM treatment for SSc, which provided a valuable clue to find novel drug targets and develop new medications for SSc.

## Abbreviations

APL: Acute promyelocytic leukemia; B: Bleomycin; Col: Collagen; CTGF: Connective tissue growth factor; ECM: Extracellular matrix; HE: Hematoxylin and eosin; N: Non-treated; NF: Normal fibroblast; P: Prevention; P & T: Prevention & treatment; Sa: Saline; SF: SSc fibroblast; siRNA: Small interfering RNA; SPARC: Secreted protein, acidic and rich in cysteine; SSc: Systemic sclerosis; T: TGF-β1; TCM: Traditional Chinese medicine; TGF-β: Transforming growth factor beta; Y: Yiqihuoxue.

## Competing interests

The authors declare that they have no competing interests.

## Authors’ contributions

JW and WW designed the study. TW carried out the *in vitro* study and its corresponding molecular studies. HC carried out the animal study and its corresponding molecular studies, as well as histopathological examinations. YM helped the *in vitro* study. QL helped the animal study. WT provided the Yiqihuoxue formula. WT, WW, MS, DC, JY and LY provided skin tissues from SSc patients and normal controls. TW, HC and JW wrote the manuscript. JW, XZ, LJ and HZ revised the manuscript. All authors read and approved the final manuscript.

## Pre-publication history

The pre-publication history for this paper can be accessed here:

http://www.biomedcentral.com/1472-6882/14/224/prepub

## Supplementary Material

Additional file 1: Figure S1 Relative activity of SBE and phosphorylation of Smad3 in NIH/3T3 fibroblasts with different treatments. **(S1)** Relative activity of SBE in the region of type I collagen promoter in murine 3T3 fibroblasts with different treatments. N, non-treated; T, TGF-β1; Y, whole Yiqihuoxue formula; Y^−^, Yiqihuoxue formula without *Tuyuan*; Y^−1^, Yiqihuoxue formula without *Agkistrodon piscivorus*; Y^−2^, Yiqihuoxue formula without both *Tuyuan* and *Agkistrodon piscivorus*. Bars showed the mean ± SD results of three assays. *, *P* <0.05. **(S2)** Western blotting analysis of p-Smad3 and Smad3 in murine 3T3 fibroblasts with different treatments. Click here for file
