# Supplementary figures and images for: Dissection of the mechanism of traditional Chinese medical prescription-Yiqihuoxue formula as an effective anti-fibrotic treatment for systemic sclerosis
Source: BMC Complement Altern Med. 2014 Jul 7;14:224. doi: 10.1186/1472-6882-14-224 (PMC4226964; doi:10.1186/1472-6882-14-224)

S1

Relative luciferase activity

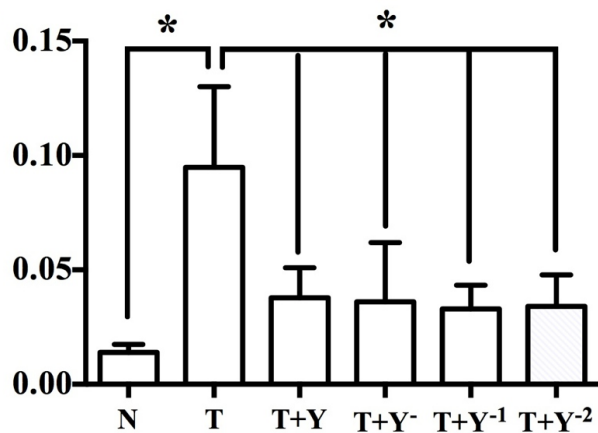

S2

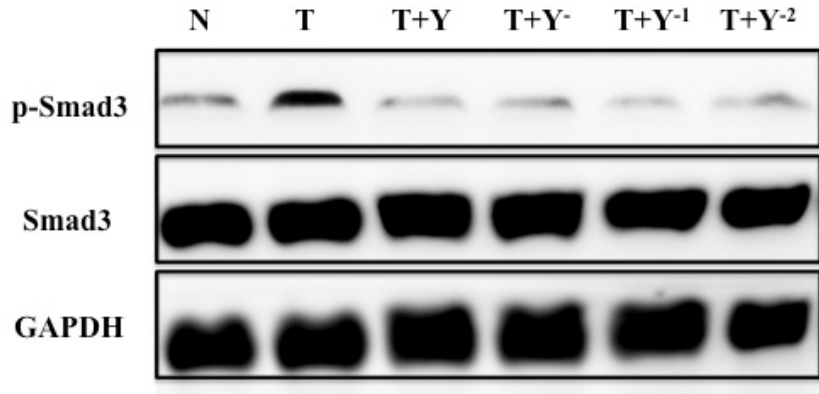

Supplement: Additional file 1: Figure S1 — Relative activity of SBE and phosphorylation of Smad3 in NIH/3T3 fibroblasts with different treatments. (S1) Relative activity of SBE in the region of type I collagen promoter in murine 3T3 fibroblasts with different treatments. N, non-treated; T, TGF-β1; Y, whole Yiqihuoxue formula; Y−, Yiqihuoxue formula without Tuyuan; Y−1, Yiqihuoxue formula without Agkistrodon piscivorus; Y−2, Yiqihuoxue formula without both Tuyuan and Agkistrodon piscivorus. Bars showed the mean ± SD results of three assays. *, P <0.05. (S2) Western blotting analysis of p-Smad3 and Smad3 in murine 3T3 fibroblasts with different treatments. [file 1472-6882-14-224-S1.pdf]
